# mTORC2 Is Required for Rit-Mediated Oxidative Stress Resistance

**DOI:** 10.1371/journal.pone.0115602

**Published:** 2014-12-22

**Authors:** Weikang Cai, Douglas A. Andres

**Affiliations:** Department of Molecular and Cellular Biochemistry, University of Kentucky, Lexington, Kentucky, United States of America; Institute of Biosciences and Technology, Texas A&M Health Sciences Center, United States of America

## Abstract

Rit, a member of the Ras family of GTPases, has been shown to promote cell survival in response to oxidative stress, in part by directing an evolutionarily conserved p38 MAPK-Akt survival cascade. Aberrant Rit signaling has recently been implicated as a driver mutation in human cancer, adding importance to the characterization of critical Rit effector pathways. However, the mechanism by which Rit-p38 signaling regulated Akt activity was unknown. Here, we identify mTORC2 as a critical downstream mediator of Rit-dependent survival signaling in response to reactive oxygen species (ROS) stress. Rit interacts with Sin1 (MAPKAP1), and Rit loss compromises ROS-dependent mTORC2 complex activation, blunting mTORC2-mediated phosphorylation of Akt kinase. Taken together, our findings demonstrate that the p38/mTORC2/Akt signaling cascade mediates Rit-dependent oxidative stress survival. Inhibition of this previously unrecognized cascade should be explored as a potential therapy of Rit-dependent malignancies.

## Introduction

Reactive oxygen species (ROS) stimulate signaling pathways that influence diverse cellular processes, from survival to aging [1]. However, the generation of excessive ROS results in oxidative stress and leads to molecular and cellular damage, contributing to the pathogenesis of numerous human diseases. ROS-activated signaling pathways have evolved to promote cell survival and homeostasis, by coupling stress stimuli to appropriate cellular responses. A balance must be maintained between pathways that promote survival or death. Exposure of cells to oxidative damage induces activation of numerous intracellular signaling pathways, including cascades controlled by the p38 MAPK, also known as stress-activated protein kinase [2]. We recently identified a fundamental role for the Rit GTPase in the regulation of survival in cells adapting to oxidative stress. Rit was found to direct an evolutionarily conserved p38-MK2-HSP27-Akt cascade, although the mechanism of p38-dependent Akt regulation remained elusive [3]. Akt is a central signaling kinase that coordinates multiple signal transduction cascades to control cell proliferation, metabolism, and survival [4]. Akt is activated by two sequential phosphorylation events. The first, by PDK1, occurs on the activation loop (Thr-308 in Akt1) within the catalytic domain, resulting in partial Akt activation [4]. Maximal catalytic activity requires a second phosphorylation event, at Ser-473 in the Akt hydrophobic domain. Recent studies have identified the mTOR complex 2 (mTORC2) as the kinase responsible for Akt Ser-473 phosphorylation [5] but it also directs Akt Thr-450 phosphorylation to regulate protein folding and stability [6], [7].

The mammalian target of rapamycin (mTOR) is an evolutionarily conserved serine/threonine kinase which functions to control fundamental aspects of cellular metabolism, growth, differentiation, and survival [8]. The mTOR pathway involves two signaling complexes with distinct regulatory and cellular activity (mTOR complex 1 and 2). mTORC1 is distinguished by the mTOR-associated adaptor protein Raptor, its greater sensitivity to rapamycin, and central role in the control of protein synthesis and cellular metabolism. The mTORC2 complex contains Rictor and Sin1 as core components, is essential for cell viability, and is a critical regulator of Akt signaling, but is also involved in the control other AGC family kinases [9] and has been found to regulate actin cytoskeleton dynamics, motility, and chemotaxis in a variety of cell types [10], [11].

Here, we present evidence that Rit interacts with the mTORC2 complex through Sin1 (stress-activated protein kinase (SAPK)-interacting protein 1). Loss of Rit protein, or inhibition of p38 MAPK signaling, blunts hydrogen peroxide-, but not mitogen-mediated activation of both mTORC2 and its downstream target Akt, serving to sensitize cells to oxidative damage. Disruption of mTORC2 by RNAi-mediated interference methods blocks Rit-dependent Akt activation, without altering Rit-p38/MK2 signaling, and results in increased cellular ROS sensitivity. These results suggest a model in which mTORC2 is an essential component in Rit-p38-dependent survival signaling in response to oxidative stress, and that p38/MK2 kinases function as upstream regulators of mTORC2 signaling in cells adapting to oxidative damage.

## Materials and Methods

### Plasmids and Reagents

Human Akt was subcloned into pEBG (J. H. Kehrl, NIAID, NIH) and flag-tagged dominant negative p38α was kindly provided by Dr. J. Han (Xiamen University, China). Lentivirus packaging vector pSPAX2, lentivirus envelop vector pMD2.G, and lentiviral shRNA expressing vector containing shRNA sequences for mTORC1/2 components (pLKO.1-shCTR, shRictor, shSin1, shRaptor) were provided by Dr. T. Gao (University of Kentucky, Lexington KY). Lentivirus was produced by the Department Molecular and Cellular Biochemistry Viral Core (University of Kentucky, Lexington KY).

Commercial antibodies were used: Flag, β-actin (Sigma); phospho-p38, p38, phospho-ERK1/2, ERK1/2, phospho-Akt (Ser473 and Thr308), Akt, phospho-MK2 (Thr334), MK2, phospho-HSP27 (Ser82), Rictor, mTOR, Raptor (Cell Signaling); Rictor (Bethyl); and HSP27, Sin1 (CalBiochem).

### Cell Culture, Transfection and Infection

Wild-type and Rictor^-/-^ mouse embryonic fibroblasts (MEFs) were the kind gift of Dr. T. Gao (University of Kentucky, Lexington). MEFs and HeLa cells were maintained in Dulbecco's modified Eagle's medium (DMEM, Gibco) supplemented with 10% heat-inactivated fetal bovine serum (FBS, HyClone), 100 U/ml penicillin and 100 µg/ml streptomycin (Gibco), and cultured at 37°C in a humidified atmosphere of 5% CO_2_. HeLa cells were transfected using Transgin (Apharma) according to the manufacturer's protocol. For lentiviral infection, HeLa cells (8×10^5^ per 60 cm dish) were incubated (5 h) with lentivirus (5 µl) with 10 µg/ml polybrene (Sigma), re-fed and incubated overnight. Forty-eight hours post-infection cells were selected with puromycin (2 µg/ml) for 7 days before proceeding to other assays.

### Co-Immunoprecipitation

To examine protein interactions by co-immunoprecipitation (Co-IP), cell lysates were prepared from HEK-293T cells transiently co-transfected with epitope-tagged protein expression vectors (GFP-EV, GFP-Sin1, HA-EV, HA-Rit^Q79L^ or HA-K-Ras^Q61L^ as indicated). Cells were allowed to recover for 48 h and then harvested using lysis buffer [20 mM Hepes (pH 7.4), 150 mM NaCl, 50 mM KF, 50 mM β-glycerolphosphate, 2 mM EGTA (pH 8.0), 1 mM Na_3_VO_4_, 1% Triton X-100, 10% glycerol, and 1× protease inhibitor cocktail]. 400 µg protein lysates were incubated with 2 µg of the required antibody (anti-HA or GFP antibody as indicated) in a total volume of 800 µL for 1 h at 4°C with end-to-end rotation. The immunocomplexes were affinity absorbed onto 15 µL of protein G-sepharose beads (GE Healthcare) for an additional hour at 4°C with end-to-end rotation. The Sepharose resin was washed sequentially: 1 time with lysis buffer, 2 times with lysis buffer containing 500 mM NaCl, and 2 times with kinase lysis buffer. Bound proteins were eluted by incubation for 5 min at 100°C in SDS loading buffer. The bound proteins along with 25 µg total cell lysates from each sample were resolved using SDS-PAGE, transferred to nitrocellulose membranes and subjected to immunoblotting using the indicated antibodies including biotinylated-HA antibody for HA-tagged Rit^Q79L^ or K-Ras^Q61L^ detection, and detected by chemiluminescence following addition of the appropriate horseradish peroxidase-conjugated secondary antibody or horseradiash peroxidase-conjugated streptavidin (Zymed) as described [12].

### Cell Viability Analysis

HeLa cells were plated in 96-well plates (4,000 cells per well) and allowed to recover for 24 h. On the day of the experiment, cells were left untreated or subjected to the indicated treatment. CellTiter 96 Aqueous Non-Radioactive Cell Proliferation Assay kit (Promega, Madison, WI) was used to measure cell viability according to manufacturer's protocol.

### Protein Phosphorylation Analysis

Cells were starved in serum free DMEM for 3–5 h prior to H_2_O_2_, EGF or IGF-1 stimulation. Whole cell lysates were prepared using kinase lysis buffer [20 mM Hepes (pH 7.4), 150 mM NaCl, 50 mM KF, 50 mM β-glycerolphosphate, 2 mM EGTA (pH 8.0), 1 mM Na_3_VO_4_, 1% Triton X-100, 10% glycerol, and 1× protease inhibitor cocktail (CalBiochem)] and subject to phospho-specific immunoblotting.

### mTORC2 Complex Kinase Assay

mTORC2 activity was examined using an *in vitro* kinase assay with inactive His-Akt (Millipore) as substrate [5]. Briefly, HeLa cells were treated as indicated and harvested using mTORC2 lysis buffer [40 mM Hepes (pH 7.4), 120 mM NaCl, 50 mM KF, 10 mM β-glycerolphosphate, 1 mM EDTA (pH 8.0), 1 mM Na_3_VO_4_, 0.3% CHAPS, and 1× protease inhibitor cocktail]. Protein lysates (1 mg) were subject to anti-Rictor (1 µg) immunoprecipitation (90 min, 4°C with end-to-end rotation, total volume 600 µL), captured with protein G-sepharose resin (15 µL) (GE Healthcare) (1 h, 4°C with end-to-end rotation), washed 4 times with mTORC2 kinase lysis buffer, and twice with mTORC2 kinase buffer [25 mM Hepes (pH 7.4), 100 mM KAc, and 1 mM MgCl_2_]. Affinity resin was incubated in kinase assay reaction mix (15 µL) [TORC2 kinase buffer containing 200 ng inactive His-Akt and 200 µM ATP] for 30 min at 37°C and the reaction terminated by incubation for 5 min at 95°C following addition of kinase buffer (135 µl) and 4× SDS sample buffer (50 µl). Recombinant His-Akt migrates differently than endogenous Akt (not shown), ensuring that this assay monitors exogenous, rather than endogenous levels of Akt phosphorylation. Samples (20 µl) were resolved on 10% SDS-PAGE and kinase activity monitored by immunoblotting with phospho-Akt (Ser473) specific antibody. Equal loading of was confirmed by immunoblotting with the indicated antibodies.

### Statistical Analysis

All data are presented as mean + SEM. Standard Student's *t*-test or one-way ANOVA followed by Newman-Keuls *post-hoc* test was performed when appropriate to analyze the differences between the individual experiments with statistical significance set as p≤0.05.

## Results

### Rit Associates with Sin1

**Figure 1:**
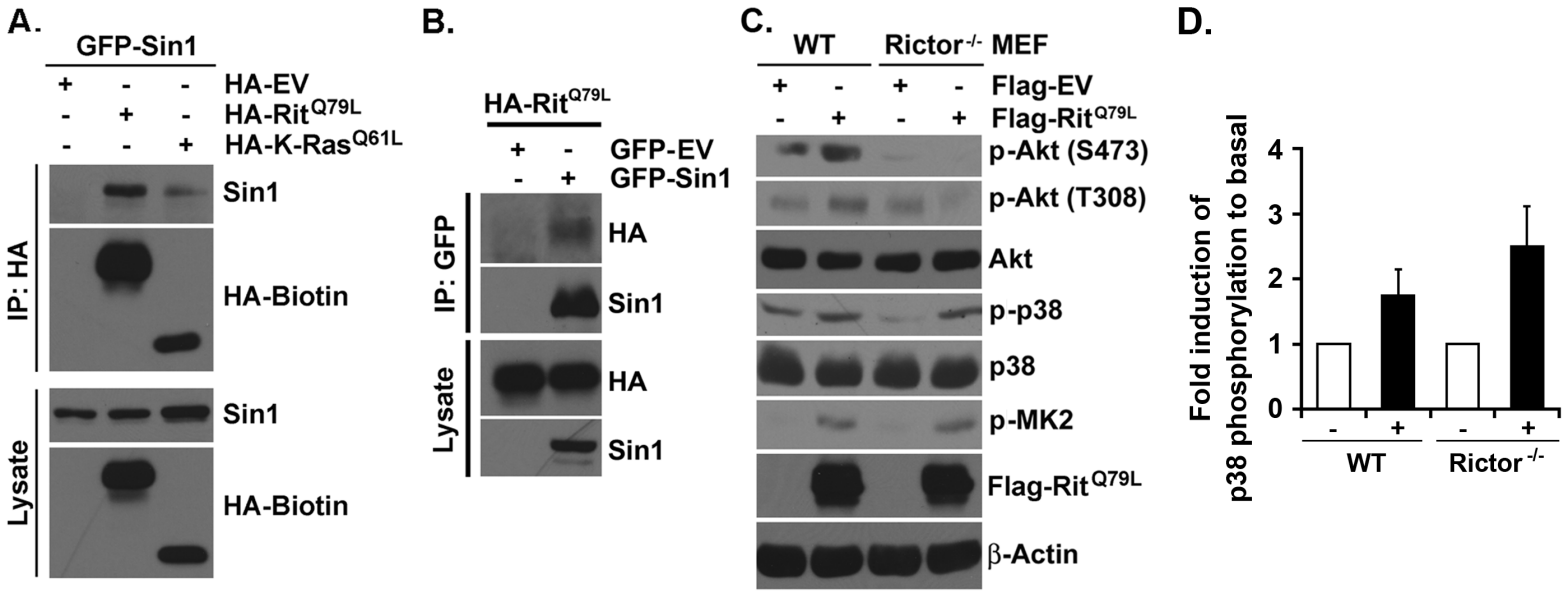
mTORC2 associates with Rit and is required for Rit-mediated Akt phosphorylation. (A) HA-Rit^Q79L^ or HA-K-Ras^Q61L^ containing complex was immunoprecipitated by HA antibody and blotted for Sin1 and Biotin-HA. (B) GFP-Sin1 containing complex was immunoprecipitated by GFP antibody and blotted for HA-Rit^Q79L^ and Sin1. (C) Lysates from serum-starved WT or Rictor^-/-^ MEFs overexpressing Flag-EV or Flag-Rit^Q79L^ were analyzed by immunoblotting with indicated antibodies. (D) Relative phosphorylation of p38 MAPK in WT and Rictor^-/-^ MEFs overexpressing Flag-EV or Flag-Rit^Q79L^. The results are presented as mean ± SEM (One-way ANOVA, n = 3).

Sin1 is required for mTORC2 formation and activity [13] and has been found to interact with K-Ras [14]. Since Rit and Ras GTPases share closely related G2 effector domains, and we have previously identified common cellular effector targets for Rit and Ras [15], we asked whether Rit could associate with Sin1. In agreement with earlier studies, GFP-tagged Sin1 co-immunoprecipitated with HA-tagged active K-Ras (K-Ras^Q61L^) when co-expressed in HEK293 cells (Fig. 1A). Sin1 also bound active Rit (Rit^Q79L^), with reciprocal immunoprecipitation confirming the interaction between active Rit and Sin1 (Fig. 1A, B).

### mTORC2 is Required for Rit-dependent Akt Activation

While Rit regulates an evolutionarily conserved p38-Akt survival cascade, the molecular mechanism directing p38-dependent Akt activation remains poorly characterized [3], [16]. Since Akt is a well-characterized mTORC2 substrate [6], [7], we examined whether mTORC2 was required for Rit-mediated Akt activation by transfecting wild-type (WT) and Rictor null (Rictor^-/-^) mouse embryonic fibroblasts (MEFs) with either empty vector or a Flag-Rit^Q79L^ expression vector (Fig. 1C). Immunblotting was used to confirm the loss of endogenous Rictor (S1 Fig.). In agreement with earlier studies [3], [16], expression of active Rit resulted in p38, MK2, and Akt activation as monitored by phospho-specific immunoblotting. However, while expression of Rit^Q79L^ resulted in elevated levels of phospho-p38 and MK2 in Rictor^-/-^ MEFs, p38-MK2 cascade activation failed to promote Akt-S473 phosphorylation (Fig. 1C, D). Taken together, these data indicate that Rit-mediated Akt activation requires mTORC2 function.

### ROS-mediated Akt Activation Requires mTORC2, but not mTORC1

**Figure 2:**
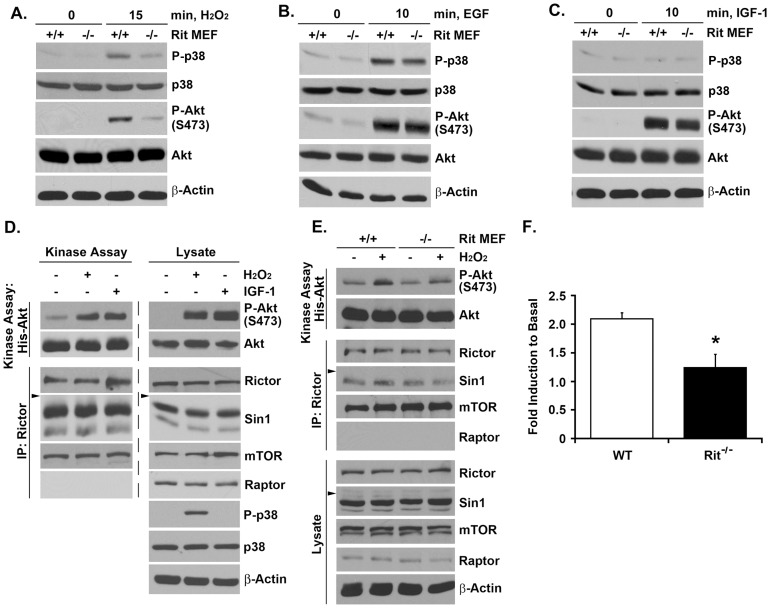
Rit mediates mTORC2 activation following oxidative stress. (A–C) Lysates from primary WT or Rit^-/-^ MEFs were prepared following H_2_O_2_ (100 µM, 15 min), EGF (100 ng/mL, 10 min) or IGF-1 (100 ng/mL, 10 min) stimulation and analyzed by immunoblotting with the indicated antibodies. (D) mTORC2 complex was immunoprecipitated using anti-Rictor antibody and subjected to *in vitro* kinase assay using inactive His-Akt as the substrate following H_2_O_2_ (1 mM, 15 min) or IGF-1 (100 ng/mL, 10 min) stimulation (*Top left*). The specificity and equal loading of Rictor immunoprecipitates was confirmed by immunoblotting with the indicated antibodies (*Bottom left*). Whole cell lysates were also analyzed by immunoblotting with the indicated antibodies (*Right*). (Arrowhead, 82kDa) (E) Anti-Rictor immunoprecipitation was used to isolate the mTORC2 complex from primary WT or Rit^-/-^ MEFs following H_2_O_2_ (100 µM, 15 min) exposure and subjected to *in vitro* kinase assay using inactive His-Akt as the substrate (Arrowhead, 82 kDa). (F) Fold induction of mTORC2 kinase activity in WT and Rit^-/-^ MEFs after stimulation relative to basal. The results are presented as mean ± SEM (*t*-test; * p<0.05, n = 3).

Rit-mediated signaling has been shown to play a critical role in the survival of MEFs in response to oxidative stress [16]. Stimulation of wild-type MEFs with hydrogen peroxide resulted in robust Akt and p38 MAPK activation, but levels of phosphorylated Akt-S473 and p38 were blunted in Rit^-/-^ MEFs (Fig. 2A). In keeping with our earlier studies [3], [16], Rit was not required for either EGF or IGF-1 mediated Akt-S473 phosphorylation (Fig. 2B, C), suggesting a privileged role for Rit in ROS-dependent, but not in growth factor-mediated, Akt signaling. To extend this analysis, the studies were repeated using a coupled kinase assay, in which the ability of immunoprecipitated mTORC2 to phosphorylate recombinant His-Akt provides a more direct measure of mTORC2 activity [5]. As seen in Fig. 2D, both exposure to hydrogen peroxide or IGF-1 results in mTORC2 activation as monitored by levels of phosphorylated recombinant Akt following anti-Rictor directed immunoprecipitation of the complex from HeLa cells. However, hydrogen peroxide-mediated mTORC2 activation was significantly reduced in Rit^-/-^ MEFs (Figs. 2E, F). These data suggest that ROS-mediated mTORC2 activation relies in part on Rit function.

**Figure 3:**
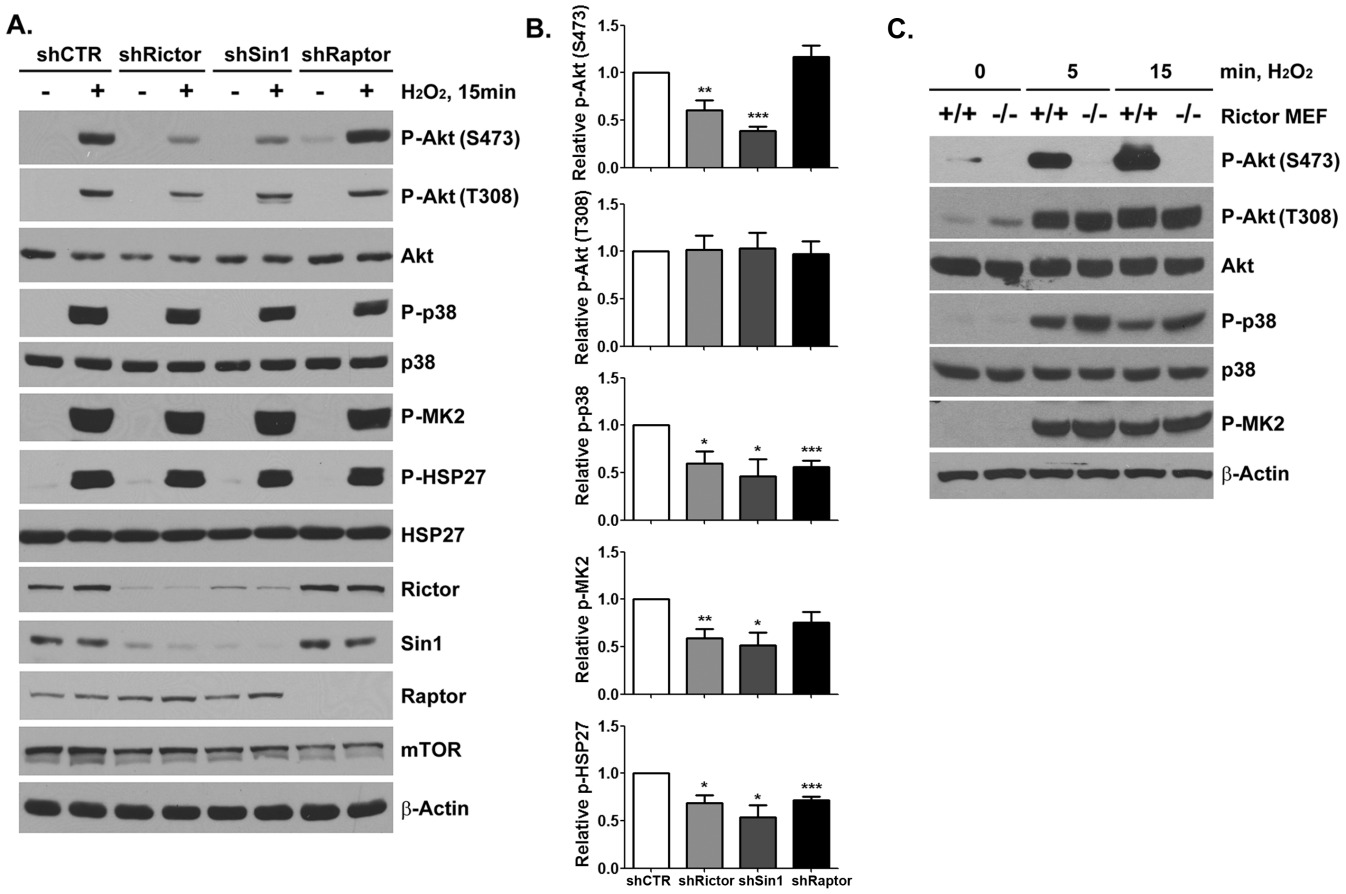
mTORC2 is required for H_2_O_2_-induced phosphorylation of Akt at Ser^473^. (A) Lysates from HeLa cells infected with lentivirus expressing shRNA against Rictor, Sin1 or Raptor were analyzed by immunoblotting with indicated antibodies following H_2_O_2_ (1 mM, 15 min) exposure (Arrowhead, 82 kDa). (B) Relative phosphorylation of signaling proteins following H_2_O_2_ stimulation. The results are presented as mean ± SEM. (*t*-test; * p<0.05, ** p<0.01, *** p<0.001 vs shCTR, n = 4). (C) Lysates from wild-type or Rictor^-/-^ MEFs were prepared following H_2_O_2_ (100 µM) treatment for indicated times and analyzed by immunoblotting with the indicated antibodies.

mTOR exists as the catalytic subunit of two biochemically and structurally distinct protein complexes, mTORC1 and mTORC2, which are distinguished by the inclusion of the mTOR-associated adaptor proteins Raptor (regulatory associated protein of mTOR) and Rictor (rapamycin-insensitive companion of mTOR) [8]. Lentiviral shRNA-mediated silencing of either Rictor (shRictor) or Sin1 (shSin1) in HeLa cells, but not Raptor (shRaptor) resulted in a significant inhibition of hydrogen peroxide-mediated stimulation of phospho-Akt-S473 levels (Fig. 3A, B). Silencing of either mTORC1 or mTORC2 complexes resulted in a decline in H_2_O_2_-mediated ctivation of the upstream p38-MK2-HSP27 cascade but phospho-Akt-T308 levels were not significantly reduced by the loss of mTORC2 function (Fig. 3A, B). Similar results were observed using knockout MEFs, with hydrogen peroxide exposure resulting in similar activation kinetics for p38, MK2, and Akt-T308 in WT and Rictor^-/-^ MEFs. However, mTORC2 complex activity was essential for hydrogen peroxide-mediated Akt-S473 phosphorylation (Fig. 3C). As expected, Raptor silencing, which disrupts mTORC1, but not mTORC2 complex activity, did not alter hydrogen peroxide-mediated Akt-S473 phosphorylation (Fig. 3A, B). Taken together, these data suggest that mTORC2 is activated in response to ROS and plays a critical role in Akt activation.

### p38 MAPK Signaling is Critical for ROS-dependent mTORC2 Activation

**Figure 4:**
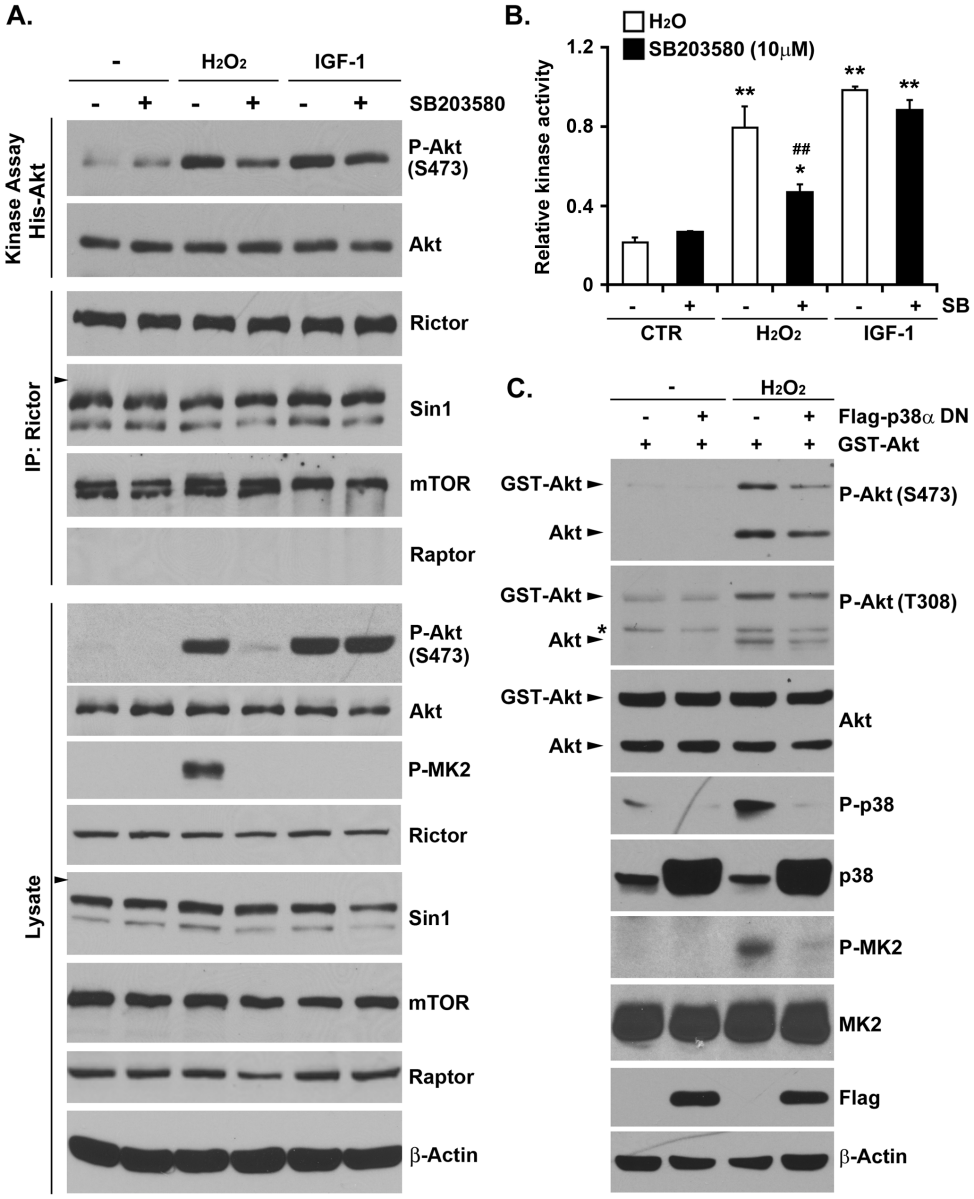
p38 MAPK controls mTORC2 kinase activity in response to oxidative stress. (A) HeLa cells were treated with 10 µM SB203580 for 30 min prior to H_2_O_2_ (1 mM, 15 min) or IGF-1 (100 ng/mL, 10 min) stimulation. mTORC2 complex was pulled down by anti-Rictor antibody and subjected to *in vitro* kinase assay using inactive His-Akt as the substrate (*Top*). The specificity and equal loading of Rictor immunoprecipitates were confirmed by immunoblotting with indicated antibodies (*Middle*). The whole cell lysates were also analyzed by immunoblotting with indicated antibodies (*Bottom*). (Arrowhead, 82 kDa) (B) Quantification of relative mTORC2 kinase activity. The results are presented as mean + SEM. (One-way ANOVA followed by Newman-Keuls *post-hoc* test when appropriate; * p<0.05, ** p<0.01 vs corresponding control groups, ## p<0.01 vs corresponding non-SB203580 treated groups, n = 3) (C) HeLa cells transfected with Flag-EV or dominant negative p38α along with GST-Akt were treated with H_2_O_2_ (1 mM, 15 min). The whole cell lysates were prepared and analyzed by immunoblotting using indicated antibodies. (*, non-specific band)

mTORC2 activation is commonly associated with growth factor signaling [8], [9]. To ascertain the importance of p38 to mTORC2 signaling in response to oxidative stress, we treated HeLa cells with the p38 inhibitor SB203580 (10 µM) and monitored mTORC2 activation following hydrogen peroxide and IGF-1 stimulation. As seen in Fig. 4A, both stimuli resulted in potent mTORC2 activation as monitored by either coupled kinase analysis (*top panel*) or phospho-Akt-S473 immunoblotting (*bottom panel*). However, SB203580 exposure resulted in the selective blunting of hydrogen peroxide-mediated mTORC2 activation (Fig. 4B). Likewise, expression of a dominant-negative p38α mutant was sufficient to diminish hydrogen peroxide-mediated Akt activation (Fig. 4C). Taken together, these data suggest that p38 plays an important role in ROS-mediated mTORC2 signaling.

### mTORC2 is Required for Cell Survival in Response to Oxidative Stress

**Figure 5:**
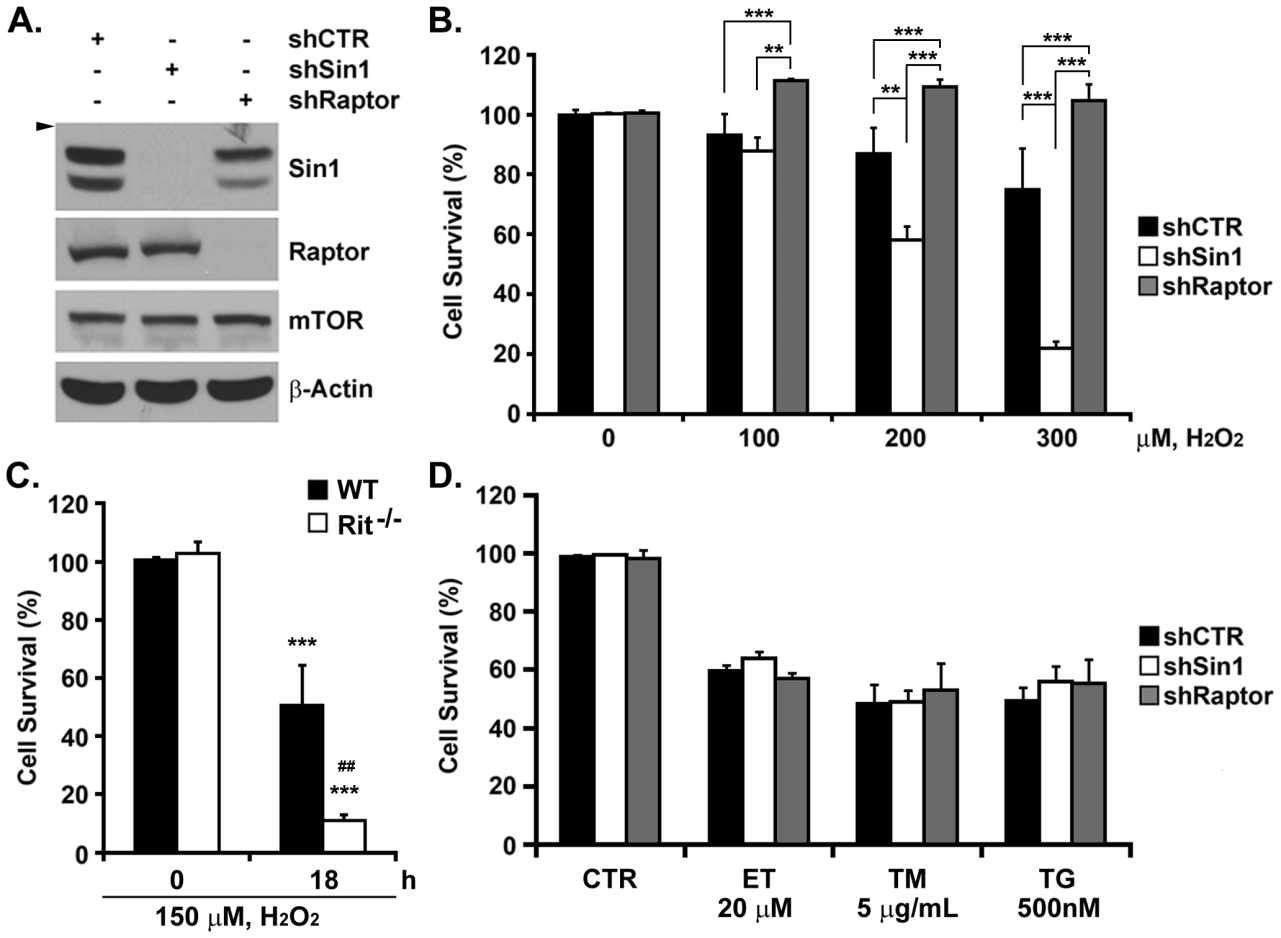
mTORC2, but not mTORC1, contributes to cell survival in response to oxidative stress. (A) Lysates from HeLa cells infected with lentivirus expressing shRNA against either Sin1 or Raptor were analyzed by immunoblotting with indicated antibodies. (Arrowhead, 82 kDa) (B) Cell viability of HeLa-shCTR, HeLa-shSin1 and HeLa-shRaptor was determined by MTS assay 24 h after the initial H_2_O_2_ exposure. The results are presented as mean + SEM. (One-way ANOVA followed by Newman-Keuls *post-hoc* test when appropriate; ** p<0.01, *** p<0.001, n = 3). (C) Cell viability of WT and Rit^-/-^ MEFs was determined by MTS assay 18 h after the initial H_2_O_2_ (150 µM) exposure. The results are presented as mean + SEM. (One-way ANOVA followed by Newman-Keuls *post-hoc* test when appropriate; ** p<0.01, n = 3). (D) Cell viability of HeLa-shCTR, HeLa-shSin1 and HeLa-shRaptor was determined by MTS assay 24 h after the initial ET (20 µM), TM (5 µg/mL) or TG (500 nM) exposure. The results are presented as mean + SEM (One-way ANOVA followed by Newman-Keuls *post-hoc* test when appropriate; n = 3).

Recent studies have implicated Rit-p38-Akt signaling as an important survival mechanism for cells responding to oxidative stress, with inhibition of this pathway sensitizing MEFs to oxidative stress, but not to DNA damage or ER stress [3], [16]. If mTORC2 activity was central to this cascade, we reasoned that mTORC2 blockade (Fig. 5A) should increase oxidative stress-dependent cell death. As shown in Fig. 5B, shRNA-mediated mTORC2 silencing (via shSin1), but not mTORC1 blockade (shRaptor), resulted in a dramatic reduction in cell viability, rivaling that seen in Rit^-/-^ MEFs (Fig. 5C), following hydrogen peroxide exposure. Importantly, this was not the case for shRNA treated cells exposed to either etoposide (20 µM), tunicamycin (5 µg/ml), or thapsigargin (500 nM) (Fig. 5D), in which shRNA control, mTORC1, or mTORC2-treated cells, displayed equivalent reductions in cell viability.

## Discussion

We report here that mTORC2 signaling plays an essential role in Rit-mediated Akt activation in response to oxidative stress. Rit interacts with Sin1 *in vitro*, and in the absence of Rit, H_2_O_2_-mediated mTORC2 and Akt activity is blunted. Importantly, Rit is not required for growth factor-mediated mTORC2-Akt signaling. Consistent with earlier studies in which p38 was found to be critical for Rit-dependent Akt activation [3], [16], inhibition of p38 MAPK signaling blunts ROS-mediated mTORC2 activity. Together these data identify mTORC2 as a critical downstream component of Rit survival signaling, serving as an essential element in the evolutionarily conserved Rit-p38-Akt survival signaling, and strongly implicate p38 in ROS-mediated mTORC2 activity. The results also provide a means to selectively blunt ROS-dependent mTORC2 activation, allowing for future analysis of the contribution of mTORC2 signaling in the survival of cells responding to oxidative stress.

mTOR kinase activation is controlled by two primary regulatory mechanisms: phosphorylation of proteins within the larger scaffolded complex by cellular kinase cascades, and through interactions with members of the Ras family of small GTPases [8]. For example, activation of mTORC1 by amino acids requires Rheb, but also involves a second family of GTPases, the Rag proteins, which direct mTORC1 to Rheb containing membranes [17]. In contrast to mTORC1, the regulation of mTORC2 is less well characterized, although the Rab GTPase Rhy1 has recently been shown to control TORC2-mediated activation of the AGC kinase Gad8 in *S. pombe* [18] and Rac1 has been found to directly bind mTOR in response to growth-factor stimulation, guiding mTORC1 and mTORC2 to subcellular compartments enriched in both substrates and additional activators [19]. Stimulation of TORC2-Gad8 signaling requires GTP-bound Ryh1, with Ryh1 promoting the interaction between Gad8 and the Sin1 subunit of the TORC2 complex [18]. Here we provide the following evidence that mTORC2 functions downstream of Rit to mediate Akt activation in response to oxidative stress. First, deletion of Rit in MEFs significantly reduces both mTORC2 and Akt activation following oxidative stress, but importantly does not alter growth factor-dependent activation of these kinases. The residual Akt phosphorylation observed in Rit^-/-^ MEFs implies that additional mTORC2 regulators contribute to ROS-dependent mTORC2-Akt signaling, although these cascades remain to be identified. Second, using both shRNA-mediated silencing techniques and Rictor null MEFs we demonstrate that mTORC2 activity is essential for Rit-dependent Akt activation as monitored by anti-phospho-Akt-S473 immunoblotting. Moreover, inhibitor studies suggest that p38 acts upstream of mTORC2 to regulate Akt phosphorylation in response to oxidative stress. Third, Rit interacts with Sin1, providing a means for Rit to associate with the larger mTORC2 complex. Finally, silencing of either Rit or mTORC2 results in a similar cellular phenotype, selective oxidative stress sensitivity, without significantly altering survival in response to ER-stress or DNA damaging agents, corroborating that Rit and mTORC2 are involved in a common cellular process. We have previously demonstrated that Rit associates with a HSP27-scaffolded p38-MK2-Akt signaling complex that contributes importantly to ROS-mediated Akt activation [3], [16]. Thus, the interaction of Rit with Sin1 might be an important molecular link in this cascade. We speculate that Rit serves to bridge the HSP27 and mTORC2 complexes, allowing phosphorylation of one or more members of the mTORC2 complex by the p38 or MK2 kinases associated with HSP27 while bringing active mTORC2 in proximity to HSP27-scaffolded Akt. Additional studies will be needed to explore these possibilities.

The p38 MAPK pathway is a critical evolutionarily conserved mediator of the cellular response to diverse forms of environmental stress. As one class of the stress-activated MAPKs (SAPKs), p38 kinases have been shown to phosphorylate a wide range of nuclear and cytoplasmic substrates to regulate processes as diverse as apoptosis, differentiation, proliferation, and cellular stress response [2]. Since we demonstrate that p38 inhibition blunts both mTORC2 and Akt activity in response to oxidative stress, p38 is likely to act upstream of mTORC2, but the molecular mechanism involved awaits further study. Previous work has shown that p38-MK2 signaling results in the direct phosphorylation of TSC2, leading to increased mTORC1 activity [20]. p38 kinases have also been implicated in H_2_O_2_-stimulated mTORC1 activation [21], and more recently energy-starvation-induced mTORC1 inactivation was shown to rely upon the p38β-PRAK cascade [22], although these mechanisms are unlikely to directly inform the nature of p38-dependent mTORC2 regulation. Members of the Sin1 family have been found to play evolutionarily conserved roles in the regulation of cellular stress response signaling and actin dynamics. The *Dictyostelium* Sin1 protein, RIP3, was first identified as a Ras-interacting protein required for chemotaxis [23], and more recent studies indicate that Ras GTPases serve as upstream regulators of TORC2-PKB signaling to direct cell migration and chemotaxis [24]. Deletion of Avo1p, the *S. cerevisiae* Sin1 protein, results in an actin cytoskeleton defect [25], with TOR2 playing a critical role in ROS signaling [26], and genome stability [27], to maintain cell viability. Interestingly, Sin1 was identified in a yeast two-hybrid screen as a Spc1 SAPK interacting protein [28], and subsequent studies have shown that mammalian Sin1 also binds p38 [29]. In *S. pombe*, Sin1 is an essential component of the TORC2 complex required for activation of the AGC–family kinase Gad8 [30]. Although Sin1 is not required for SAPK-regulated stress gene expression in *S. pombe*, and the functional significance of the Sin1/Spc1 interaction is unknown, strains lacking functional TORC2 or Spc1 display increased stress sensitivity [18]. This is similar to the increased sensitivity to environmental stress observed following the loss of *Drosophila* D-Ric, a Rit ortholog, a phenotype that overlaps those caused by deletion of the *D-p38a* gene [16]. The striking parallels between the regulation and physiological function of the Spc1-TOC2-Gad8 and p38-mTORC2-Akt cascades suggests that Sin1 may play an evolutionarily conserved role, coordinating p38/SAPK and TORC2 signaling to modulate cellular stress survival. Using a liver-specific *Rictor*-knockout mouse model, Lamming and colleagues recently identified an *in vivo* role for mTORC2 as an upstream regulator of hepatic p38 MAPK signaling [31]. Together, these studies suggest a complicated crosstalk between the p38 and mTORC2 pathways, although the underlying molecular mechanisms remain largely elusive and are the focus of ongoing studies.

In summary, these studies extend understanding of mTOR regulation, identifying Rit-p38 signaling as a participant in ROS-dependent mTORC2 complex activation. Understanding ROS-mediated signaling is of great importance as unregulated ROS is associated with numerous diseases, including neurodegenerative disorders, cardiovascular disease, cancer, and aging [1]. Recent work has identified somatic oncogenic mutations in Rit in a subset of lung adenocarcinoma cases [32], [33], but also in patients with myeloproliferative neoplasms, in particular, chronic myelomonocytic leukemia [34], and as gene mutated in Noonan syndrome [35]–[38]. Ectopic expression of mutated *RIT1* induces cellular transformation, identifying *RIT1* as a driver oncogene [32], [39]. Because mTORC2 activity is often elevated in cancer cells and directs metabolic reprogramming [40], [41], an intriguing possibility is that oncogenic Rit-mediated mTORC2 signaling results in the activation of pro-survival pathways. Because mTORC2 inhibitors are in development for the treatment of cancer, our findings suggest that specifically targeting p38-mTORC2 signaling could represent a therapeutic strategy for *RIT1*-mutated tumors.

## Supporting Information

S1 Fig **Deletion of Rictor reduces Sin1, but not Raptor, in MEFs.**
(TIF)
