# Supplementary figures and images for: mTORC2 Is Required for Rit-Mediated Oxidative Stress Resistance
Source: PLoS One. 2014 Dec 22;9(12):e115602. doi: 10.1371/journal.pone.0115602 (PMC4274107; doi:10.1371/journal.pone.0115602)

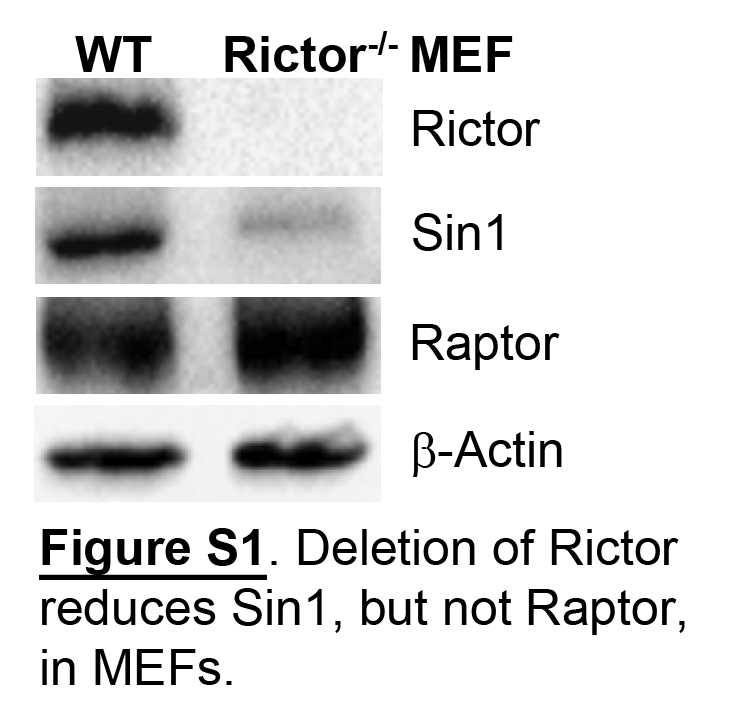

Supplement: S1 Fig — Deletion of Rictor reduces Sin1, but not Raptor, in MEFs. (TIF) [file pone.0115602.s001.tif]
